# Microbial Diversity Indexes Can Explain Soil Carbon Dynamics as a Function of Carbon Source

**DOI:** 10.1371/journal.pone.0161251

**Published:** 2016-08-23

**Authors:** Benjamin P. Louis, Pierre-Alain Maron, Safya Menasseri-Aubry, Amadou Sarr, Jean Lévêque, Olivier Mathieu, Claudy Jolivet, Philippe Leterme, Valérie Viaud

**Affiliations:** 1 UMR SAS 1069, AGROCAMPUS OUEST, Rennes, France; 2 UMR SAS 1069, INRA, Rennes, France; 3 UMR Agroecology 1347, INRA, Dijon, France; 4 Biogeosciences, University Bourgogne Franche-Comté, CNRS, Dijon, France; 5 US Infosol 1106, INRA, Ardon, France; USDA Agricultural Research Service, UNITED STATES

## Abstract

Mathematical models do not explicitly represent the influence of soil microbial diversity on soil organic carbon (SOC) dynamics despite recent evidence of relationships between them. The objective of the present study was to statistically investigate relationships between bacterial and fungal diversity indexes (richness, evenness, Shannon index, inverse Simpson index) and decomposition of different pools of soil organic carbon by measuring dynamics of CO_2_ emissions under controlled conditions. To this end, 20 soils from two different land uses (cropland and grassland) were incubated with or without incorporation of ^13^C-labelled wheat-straw residue. ^13^C-labelling allowed us to study residue mineralisation, basal respiration and the priming effect independently. An innovative data-mining approach was applied, based on generalized additive models and a predictive criterion. Results showed that microbial diversity indexes can be good covariates to integrate in SOC dynamics models, depending on the C source and the processes considered (native soil organic carbon vs. fresh wheat residue). Specifically, microbial diversity indexes were good candidates to help explain mineralisation of native soil organic carbon, while priming effect processes seemed to be explained much more by microbial composition, and no microbial diversity indexes were found associated with residue mineralisation. Investigation of relationships between diversity and mineralisation showed that higher diversity, as measured by the microbial diversity indexes, seemed to be related to decreased CO_2_ emissions in the control soil. We suggest that this relationship can be explained by an increase in carbon yield assimilation as microbial diversity increases. Thus, the parameter for carbon yield assimilation in mathematical models could be calculated as a function of microbial diversity indexes. Nonetheless, given limitations of the methods used, these observations should be considered with caution and confirmed with more experimental studies. Overall, along with other studies on relationships between microbial community composition and SOM dynamics, this study suggests that overall measures of microbial diversity may constitute relevant ways to include microbial diversity in models of SOM dynamics.

## Introduction

Among soil biological processes, mineralisation of soil organic matter (SOM) is essential, as SOM is a key component contributing to many functions and services in soil ecosystems [1,2]. Mathematical models are useful tools to quantitatively describe processes involved in SOM dynamics and help predict the influence of management practices [3]. Many SOM dynamics models have been developed to date, and microorganism biomass is increasingly represented explicitly [4]. However, microbial diversity is nearly absent in these models despite new evidence of its role in SOM dynamics. Two main reasons have led to this absence: i) from a conceptual perspective, SOM dynamics models performed sufficiently well without needing to consider microbial diversity [5]; and ii) for a long time, technical limitations have hindered demonstration of a relationship between microbial diversity and SOM dynamics, as well as identification of qualitative variables to better describe the microbial pool. The latest technical advances in molecular biology have made the latter possible, and recent results have shown that microbial diversity can significantly influence transformation of carbon (C) and nitrogen (N) in the soil [6–9]. For instance, Baumann et al. [6] found reduced lignin and wheat sugar decomposition, while Philippot et al. [8] showed reduced denitrification activity with low microbial diversity.

These results qualitatively demonstrated a microbial diversity-SOM dynamics relationship. However, there is still a need to quantitatively describe this relationship between microbial diversity and SOM dynamics processes and make it possible to integrate it in SOM models. Increasing evidence in the literature indicates that taxonomic and functional compositions of microbial communities are strong drivers of SOM processes [10], and bacterial phyla have been identified as functional groups [11]. However, phylum is a high taxonomic rank, and members of the same phylum can exhibit different functional traits. Consequently, knowledge about microbial composition and functional traits currently remains limited, and further study is required to understand the relationship between microbial composition and C and N dynamics and identify key taxa as indicators of microbial community functional traits [12]. Given this lack of knowledge, we argue that it remains difficult to use microbial composition to build the quantitative relationships discussed above, which are necessary to parameterise predictive models. Interesting modelling approaches have integrated microbial diversity by representing different functional groups of microorganisms according to their differing affinities for organic substrates [13,14] or enzyme-production strategies [15]. However, because of the limited knowledge, the microbial groups in these models are conceptual; thus, it is challenging to validate these models with empirical data [16]. In contrast, promising demonstrations of the microbial diversity-SOM dynamics relationship have occurred using microbial diversity indexes (e.g. richness, evenness, Shannon index) to assess microbial diversity [6–9]. This suggests that overall microbial diversity indexes could be relevant covariates of SOM dynamics. Since high-throughput sequencing can now easily quantify microbial diversity in environmental samples, it is possible to assess the increase in accuracy of SOM dynamics models due to integrating microbial diversity via these covariates.

Recently, Tardy et al. [9] showed that microbial diversity explained C dynamic differently according to the quality of C substrates. In particular, they demonstrated that the importance of bacterial vs. fungal diversity may vary with the recalcitrance of C substrates, in agreement with other studies which demonstrated that fungi decompose recalcitrant substrates better than bacteria [17]. Additionally, quantitative and qualitative improvement of SOM is generally observed in agroecosystems favouring a fungal-dominated community [18]. Consequently, we hypothesise that model accuracy will improve if the microbial variables distinguish bacterial and fungal diversity, each of which influence dynamics of SOM pools differently as a function of their recalcitrance. Knowing this, it is necessary to go further by building quantitative relationships between C dynamics and bacterial and fungal diversity indexes to investigate the implication of integrating microbial diversity in SOM dynamics models.

The objective of the present study was to statistically investigate the relationships between bacterial and fungal diversity and decomposition of C pools, characterised by the pattern of CO_2_ emissions under controlled conditions. In particular, we aimed to i) confirm the relevance of microbial diversity indexes as covariates to explain variability in CO_2_ emissions, ii) build quantitative relationships between microbial diversity and CO_2_ emissions and iii) evaluate implications of integrating microbial diversity in SOM dynamics models. Twenty soils with a variety of characteristics and two different land-use histories (cropland and grassland) were incubated with and without addition of ^13^C-labelled wheat residue in soils. ^13^C-labelling allowed us to distinguish and separately analyse the relationship between bacterial and fungal diversity and mineralisation of different sources of OC (soil, wheat residue), as well as the interaction between mineralisation of these two sources (priming effect). Land-use history was taken into account because of the increasing body of evidence that these two land uses differ greatly in their influence on microbial diversity in the soil [19–22], which has consequences for C cycling [9]. Consequently, we hypothesised that studying these two land uses could reveal contrasting diversity-SOM dynamics relationships that are important to understand. Diversity of bacterial and fungal communities was characterised by high-throughput sequencing of ribosomal genes. We applied an innovative data mining approach, based on generalized additive models (GAM) [23,24] and a predictive criterion, to select covariates that better explain variability in CO_2_ emissions for each C source and explore the relative importance of bacterial and fungal diversity, along with classic soil properties which influence SOM dynamics. The GAM approach provides quantitative relationships useful for integrating microbial diversity in SOM dynamics models.

## Materials and Methods

### Data source

#### Soil samples and analyses

We considered a database of 20 agricultural soils sampled from the French Soil Quality Monitoring Network (RMQS) [25]. The 20 locations of these soils had high variability in soil properties (in particular pH and texture) for two land-use classes: cropland (10 soils) and grassland (10 soils). Croplands corresponded to monoculture systems or crop rotations with or without short-term grasslands. Grasslands corresponded to permanent or long-term grasslands (more than ten years). Concerning abiotic soil properties, texture was measured by standardized 5-fraction granulometry (NF X 31–107), soil organic carbon (SOC) content was measured by standardized dry combustion (NF ISO 10694) and pH by standardized 1:5 water suspension (NF ISO 10390) [26]. These samples were then air-dried and stored in the French national soil sample archive following the protocol described by Ratié et al. [27]. S1 Table provides more information about the locations and studied soils. The field studies were carried out on private lands where owners gave permission to conduct the soil samplings. Since no protected areas were involved, no specific permissions were required. The field studies did not involve endangered or protected species.

#### Carbon mineralisation measurements

C mineralisation was measured using microcosm respiration following the procedure of Tardy et al. [9]. Microcosms were established by placing 30 g of equivalent dry soil supplemented with sterile water to attain 60% soil water-holding capacity in 150 ml hermetically sealed plasma flasks. These microcosms were pre-incubated at 20°C for three weeks to avoid the heavy disturbance (overestimation of C mineralisation) caused by adding water after storage. Three replicates of each microcosm were then amended with ^13^C-labelled powder of wheat residues (5 mg g^-1^ dry weight of soil) while three others were not (control). Both amended and control microcosms were mechanically mixed. The 120 microcosms (20 locations × 2 treatments (control and amended) × 3 replicates) were incubated in the dark for 80 days under controlled temperature (20°C) and moisture conditions (60% of water-holding capacity).

Respired CO_2_ was measured after 3, 7, 14, 21, 28, 44, 60 and 80 days of incubation in microcosms. The gaseous phase was sampled in 10 ml airtight flasks to measure the CO_2_ concentration and in 12 ml airtight flasks to determine the C isotope (^13^C) abundance. The concentrations measured at each sampling date corresponded to the CO_2_ accumulated between two sampling dates. ^13^C-labelling of the plant residues allowed separating SOC (R_s_) and plant-residue (R_r_) mineralisation (μg C-CO_2_ g^-1^ soil) using mass-balance equations:

$$R_{s}+R_{r}=R_{t}\;\text{and}\;R_{s}\times A_{s}^{13}+R_{r}\times A_{r}^{13}=R_{t}\times A_{t}^{13}$$ (1)

$$R_{s}=\frac{R_{t}\times A_{t}^{13}-R_{r}\times A_{r}^{13}}{A_{s}^{13}}$$ (2)

where $A_{i}^{13},\;i=\{s,r,t\}$ is the ^13^C abundance in soil C, plant residue and total respired CO_2_ (*R*_*t*_) emitted by amended soil, respectively.

In this study, the priming effect (PE) was calculated as:

$$PE=\frac{R_{s,amended}}{R_{s,control}}$$ (3)

where *R*_*s*,*amended*_ and *R*_*s*,*control*_ are SOC mineralisation in amended and control microcosms, respectively. PE was calculated as a ratio, rather than the more traditional difference between *R*_*s*,*amended*_ and *R*_*s*,*control*_, to avoid negative values. This allowed a log-transformation to meet the assumptions of the statistical models used later.

To assess differences in C mineralisation rates between cropland and grassland, a two-way analysis of variance (ANOVA) was performed on each mineralisation kinetic rate (R_s,control_, R_s,amended_, R_r_ and PE). The model included a fixed factor, “land use”, and a random factor, “time”. Post-hoc analysis, based on Tukey’s honest significant difference test [28], was performed to assess differences between each level of both factors and their interaction.

#### Microbial biomass and diversity determination

Following Tardy et al. [9], the microbial community was analysed in each soil just after pre-incubation and before incubation. Microbial DNA was extracted from 1 g of each soil replicate using a slight modification of the ISO-10063 procedure [29]. DNA concentrations were determined and used as estimates of molecular microbial biomass [30]. After DNA purification (MinElute gel extraction kit, Qiagen, Courtaboeuf, France), bacterial and fungal diversity was determined for each soil. For bacteria, a 16S rRNA gene fragment with sequence variability and about 450 bases for 454 pyrosequencing was amplified by PCR using primers F479 and R888. For fungi, an 18S rRNA gene fragment of about 350 bases was amplified using primers FR1 and FF390. After the pyrosequencing procedure, as described in Tardy et al. [9], bioinformatic analyses were performed on the 16S and 18S rRNA gene sequences to cluster them at 95% sequence similarity into operational taxonomic units (OTU). From OTUs, quantitative diversity indexes were calculated, such as bacterial and fungal richness (number of OTUs), as well as other indexes that consider OTU abundance (Shannon index, H’; evenness, J’; and inverse Simpson index, 1/D). H’ increases with an increase in richness and the equity of OTU abundance. J’ (range = 0–1) provides information only about the equity of OTU abundance and equals the ratio of H’ to its maximum potential value (i.e. if all OTUs had the same abundance). The index 1/D gives the probability that two individuals randomly selected from a sample will belong to the same OTU; higher values indicate greater diversity. It gives more weight to the more abundant species in a sample. The addition of rare species to a sample causes only small changes in its value, contrary to H’.

A minimal dataset with the mean values of all the response variables and covariates is available in supporting information (S2 Table).

### Statistical selection of soil properties that better predict mineralisation kinetics

We assumed that mineralisation kinetics (R_s_, R_r_, PE) are influenced by soil properties. The objective was to verify whether microbial diversity parameters are as suitable for predicting C mineralisation as the abiotic soil properties frequently considered in SOM models. Using GAM [23,24], soil properties − including microbial diversity − were statistically selected to explain variability in mineralisation kinetics. Properties were selected for each mineralisation kinetic (R_s,control_, R_s,amended_, R_r_ and PE) using the *mgcv* package [31] in the free statistical software R (R Core Team) [32].

#### Theory of generalized additive models

The structure of a GAM [23,24] can be written as:

$$g\left(E\left(Y_{i}\right)\right)=\alpha +f_{1}\left(x_{1i}\right)+f_{2}\left(x_{2i}\right)+\ldots +f_{p}\left(x_{pi}\right)$$ (4)

where *Y*_*i*_ is a response variable following some exponential family distribution, *α* is the intercept of the model, and {*f*_*j*_, *j* = 1,…,*p*} are functions of the covariates *x*_*j*_. This structure allows for nonlinear dependence of the response on the covariates. As functions *f*_*j*_’s are not necessarily known, they are specified as smooth functions rather than detailed parametric ones. Smooth functions are flexible data-driven functions estimated by semi-parametric methods often using a spline basis. Nevertheless, the *f*_*j*_’s can also be specified as known functions − e.g. identity function for a linear dependence or a power-family function for a polynomial dependence − in which case the model becomes a generalized linear model. As for generalized linear models, the link function *g* allows for a non-normal distribution of *E*(*Y*_*i*_).

#### Stepwise selection of soil properties

The response variable to predict with the GAM referred to C-CO_2_ respired at each measurement date: we focused on the rate of respired C-CO_2_ (Δ*R*/Δ*t*), defined as the amount of respired C-CO_2_ divided by the number of days between two measurement dates. To avoid heteroscedasticity in models, the logarithm of this rate for the eight measurement dates was used as a response variable for mineralisation kinetics.

The covariates considered for selection with GAM included the abiotic soil properties influencing C mineralisation usually considered in SOM models, as well as microbial diversity indicators. The abiotic variables considered were texture properties (clay, silt, sand and clay + fine silt contents), SOC content, soil C:N ratio and pH. The biological covariates were microbial richness, J', H' and 1/D for both bacteria and fungi. We also included molecular microbial biomass, as it can explain C dynamics greatly, especially interactions between residue and SOC mineralisation [33,34]. One-way ANOVA was performed to assess the significance of differences between means of soil properties of cropland and grassland.

Relationships between soil properties and the response variable were built with smooth functions estimated by penalised cubic regression splines (s). However, we also allowed *f*_*j*_ to be known parametric functions such as identity (I) or polynomial of degree 2 (poly2) or 3 (poly3). Using these functions reduced the risk of overfitting, a well-known limitation of the GAM approach [31]. To assess differences in relationships with cropland and grassland soils, each combination of soil properties and functions *f*_*j*_ interacted with a covariate factor corresponding to “land use”. By doing this, cropland and grassland soils could have different relationships between soil properties and response variables.

**Fig 1:**
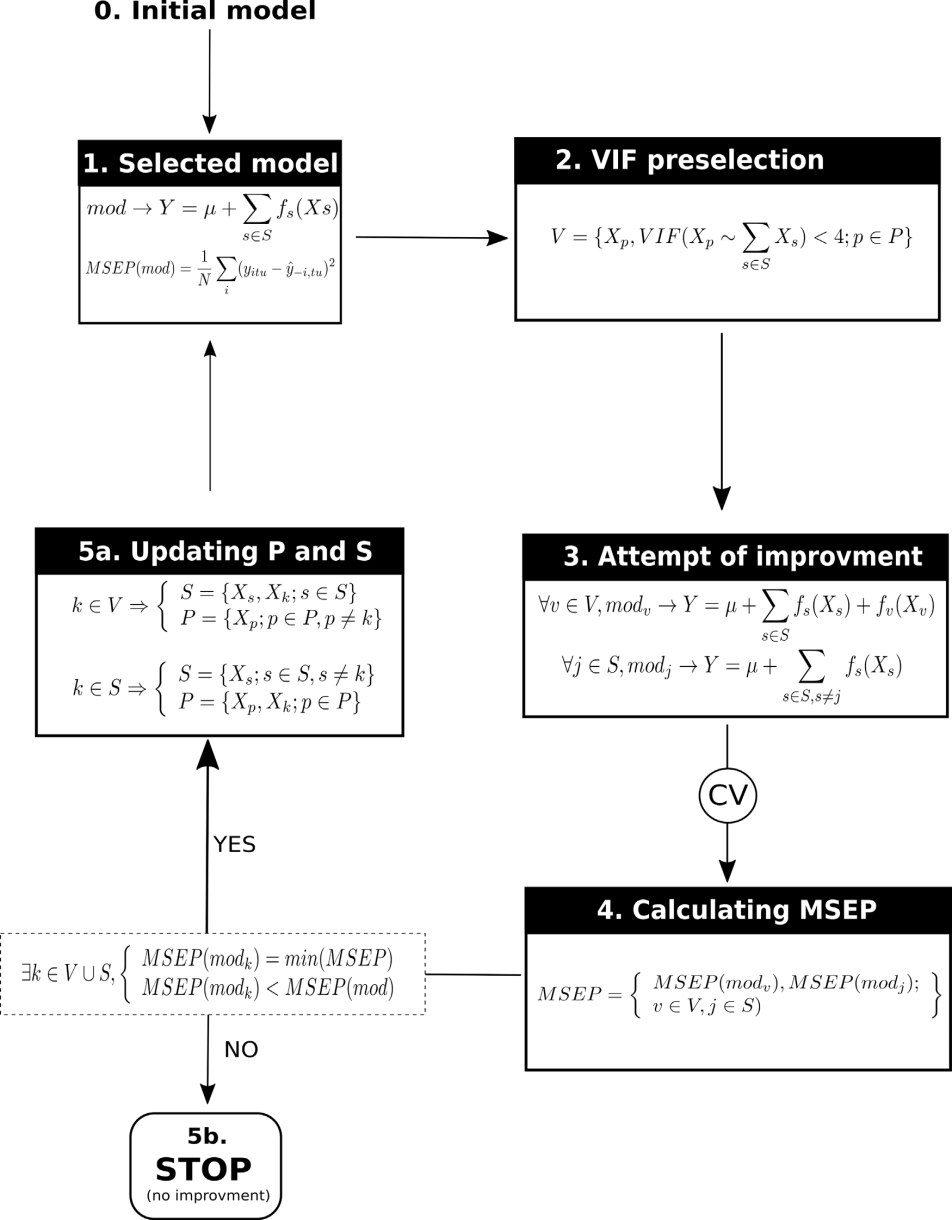
Predictor selection procedure using generalized additive models. P is the set of potential covariates, S is the set of selected covariates, V is a sub-set of P that contains potential covariates not collinear with covariates in S (i.e. already selected), MSEP() indicates the function to calculate MSEP, *fj*’s are functions (smooth or parametric) to represent relationships between covariates and the response variable (*Y*) and CV means cross validation.

Since rates of respired C were time dependent, time (used as a categorical variable) was an obligatory covariate in the GAM. Since measures in mesocosms occurred over time, the covariate “time” represents a temporal pseudo-replication and, consequently, was considered as a random factor. Consequently, the simplest model selected (Fig 1; step 0, initial model) was:

Y=μ+time (5)

where *Y* is the response variable (mineralisation kinetic), *μ* is the intercept, and *time* is the random effect of covariate time (in days). We defined P as all soil properties–which were potential covariates in GAM–and S as the soil properties selected during the selection procedure. Starting from Eq 5, the steps of the selection procedure were (Fig 1):

1. Calculate a mean squared error of prediction (MSEP) of the currently selected model by cross-validation:
  $$MSEP=\frac{1}{N}\sum_{i}{\left(y_{itu}-{\hat{y}}_{-i,tu}\right)}^{2}$$ (6)
  where *y*_*itu*_ is the value of the response variable for soil *i* at time *t* for replicate *u*, ${\hat{y}}_{-i,tu}$ is the estimated value of the response variable for soil *i* at time *t* for replicate *u* using the selected model calibrated without values of soil *i*, and *N* is the total number of values for the response variable. MSEP is a classic indicator of the predictive quality of a model. Cross-validation estimation of MSEP is known to be less subject to overfitting than a classic goodness-of-fit index.
2. To avoid problems associated with collinearity of covariates in GAM [35], pre-select soil properties based on a variance-inflation factor in linear models between each soil property in P and the soil properties already selected in the model (S) with a threshold of 4 [36].
3. Build all potential improved models by adding one of each combination of pre-selected soil properties in R and associated functions (I, poly2, poly3, and s) or by removing one previously selected soil property. The latter enabled removing previously selected soil properties in case they contained redundant information with newly selected ones.
4. Calculate MSEP for each model by cross-validation.
5. Watch for the model *k* with the smallest MSEP:
  - If the MSEP of model *k* is smaller than that of the currently selected model (step 1), model *k* becomes the new selected model. If model *k* was better because of a newly added soil property from P, this soil property becomes the one selected and moves to group S (i.e. those previously selected). If model *k* was better because of the removal of a previously selected soil property, the latter moves from group S to P. The procedure returns to step 1.
  - If the MSEP of model k is greater than that of the currently selected model (step 1), there is no more improvement, and the procedure stops. The current model is the final one, and S contains all of the pre-selected soil properties that best predict the response variable.

#### Assessment of selected models

After the selection procedure, model residuals were graphically checked for identical and independent distribution hypotheses, as GAM can be sensitive to violation of distribution assumptions [31]. The total percentage of explained deviance (%Dev) was measured to assess the goodness-of-fit of the selected models. To assess the predictive quality of the selected models, the ratio of inter-quartile range to root MSEP (RPIQ) was calculated as the ratio of the inter-quartile range (IQR) of the response variable to the square root of the MSEP calculated according to Eq 6. RPIQ, developed by Bellon-Maurel et al. [37], represents the degree to which the dispersion of the response variable exceeds the model’s prediction error. Higher RPIQ indicates better predictive quality of the model.

#### Relative importance of selected soil properties

The relative importance of selected soil properties was assessed by estimating the contribution of each selected soil property to the deviance using the R package *vegan* [38]. To achieve this, each component of the linear predictor, i.e. the transformation of each soil property by its I, poly2, poly3 or s function, was extracted from the model, and redundancy analysis was performed between the response variable and these components. Following a variance partitioning approach, the percentage of deviance explained by each component was calculated using the sum of squares from an ANOVA of the redundancy analysis result.

## Results

### Comparison of soil properties and cumulative respiration of cropland and grassland

**Fig 2:**
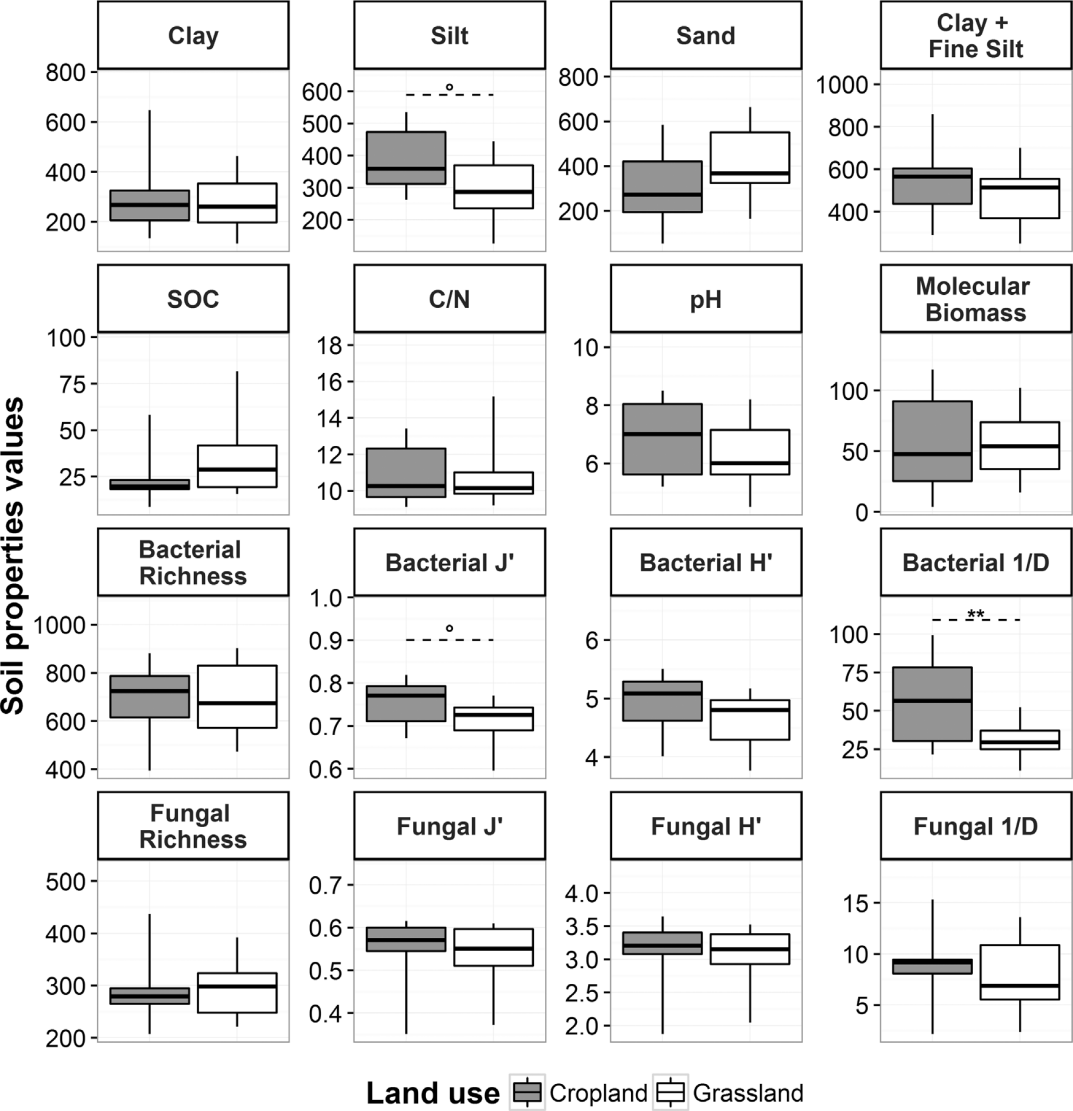
Variability in soil property values in croplands and grasslands. Bottom and top whiskers of boxplots extend to the lowest and highest values, respectively. Units of soil properties are [g.kg^-1^ soil] for texture variables and soil organic carbon (SOC) content, [μg DNA.g^-1^ soil] for molecular biomass, [number of operational taxonomic units (OTUs)] for bacterial and fungal richness, [pH unit] for pH and dimensionless for C:N ratio and bacterial and fungal Shannon index (H’), evenness (J’) and inverse Simpson index (1/D). Dashed lines above boxplots indicate significant differences between cropland and grassland, and the symbol shows the level of significance (*** P<0.001, ** 0.001<P<0.01, * 0.01<P<0.05, ° 0.05<P<0.1).

Except for bacterial 1/D, Bacterial J’ and Silt (P = 0.002, 0.079 and 0.053, respectively), soil properties did not differ significantly between cropland and grassland soils at the 10% level of significance between cropland and grassland (Fig 2). However, the dispersion of SOC content was slightly larger in grassland soils than in cropland soils, as the median in grassland soils was higher than the third quartile in cropland soils.

**Fig 3:**
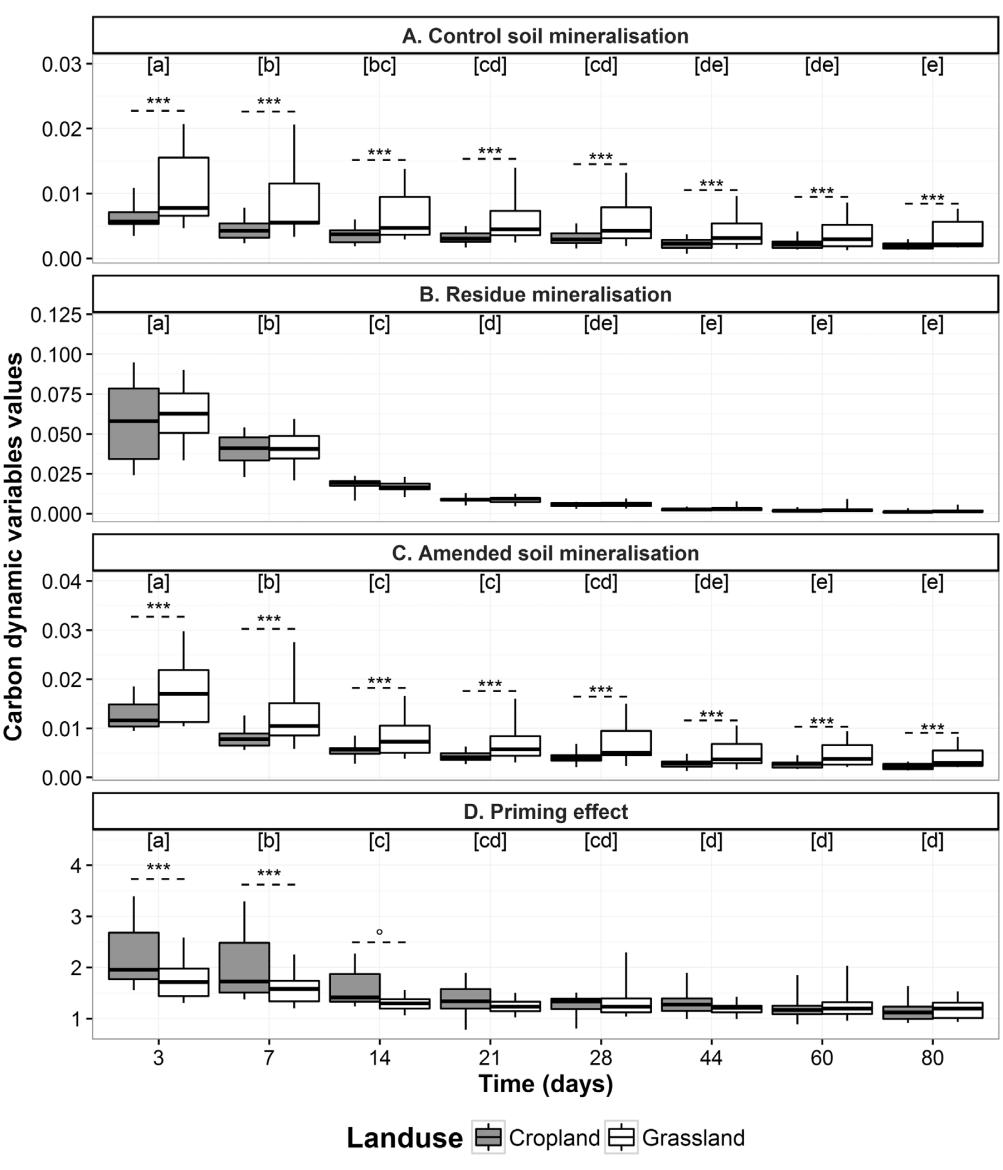
Variability in mineralisation rate and priming effect (PE) in croplands and grasslands at each sampling time. (A) Control-soil mineralisation rate (R_s,control_, mg C-CO_2_ g^-1^ soil day^-1^), (B) residue mineralisation rate (R_r_, mg C-CO_2_ g^-1^ soil day^-1^), (C) amended-soil mineralisation rate (R_s,amended_, mg C-CO_2_ g^-1^ soil day^-1^) and (D) PE (dimensionless). Bottom and top whiskers of boxplots extend to the lowest and highest values, respectively. Dashed lines above boxplots indicate significant differences between cropland and grassland, and the symbol shows the level of significance (*** P<0.001, ** 0.001<P<0.01, * 0.01<P<0.05, ° 0.05<P<0.1). Different letters above boxplots indicate significant (P<0.05) differences between incubation times.

Regarding soil functioning, ANOVA showed significant differences (P < 0.05) between incubation times (Fig 3). Significant differences (P < 0.05) were also observed between cropland and grassland at each time of incubation, except for the PE, whose difference was significant only at 3 and 7 days (P = 0.002 and 0.007, respectively) and at 14 days at the 10% level of significance (P = 0.069).

### Quality of selected models

The selected models were able to explain large percentages of the variability in mineralisation response variables, as %Dev ranged from 74% for the PE model to 93% for the residue and control mineralisation model (Table 1). For all models, a large percentage of explained deviance was due to the time covariates, ranging from 33% in control soil mineralisation to 93% in residue mineralisation. Predictive qualities of the models were more variable, ranging from moderate (RPIQ≈2) to good (RPIQ≥6). RPIQ values increased in the following order: PE (RPIQ = 2), control soil mineralisation (RPIQ = 2.9), amended soil mineralisation (RPIQ = 4), and residue mineralisation (RPIQ = 6). Model complexity had the opposite order, as assessed by the total degrees of freedom in the models.

**Table 1:** Assessment of models and covariates selected for each mineralisation parameter.

	df	Variance explained (%)	RPIQ	Covariates	Interaction^a^	Relation^b^	Significance^c^	Relative Importance^d^ (%)
**Control soil mineralisation** (R_s,control_)	18.98	93	2.9	Time	NO	RF	***	33
				Sand	NO	P3	***	8
				C:N ratio	YES	I	***	7
				Bacterial J’	YES	P3	***	44
**Amended soil mineralisation** (R_s,amended_)	13.98	90	4	Time	NO	RF	***	60
				SOC	YES	I	***	13
				pH	NO	I	***	12
				Fungal richness	NO	P2	***	4
				Fungal 1/D	NO	I	***	<1
**Residue mineralisation** (R_r_)	8.99	93	6	Time	NO	R	***	93
				Silt	NO	I	***	<1
**Priming effect** (PE)	19.98	74	2	Time	NO	RF	***	46
				Clay+Fine Silt	YES	P2	***	1
				SOC	NO	I	ns	1
				C:N ratio	YES	s	**	1
				pH	NO	I	**	13
				Molecular biomass	NO	I	**	3
				Fungal 1/D	NO	s	***	9

df, degrees of freedom; RPIQ, Ratio of inter-quartile range to root Mean Square Error of Prediction; J’, evenness; SOC, Soil Organic Carbon; 1/D, inverse Simpson index

^a^ Interaction with land-use?

^b^ Type of relationship selected: RF, random factor; I, identity; P2, polynomial of degree 2; P3, polynomial of degree 3; s, smooth function

^c^ Significance of selected covariates based on the Fisher test: *** P<0.001; ** 0.001<P<0.01; * 0.01<P<0.05; ° 0.05<P<0.1; ns (not significant) P>0.1

^d^ Relative importance of selected covariate based on variance partitioning

### Relative importance of selected soil properties in predictions of mineralisation kinetics

**Fig 4:**
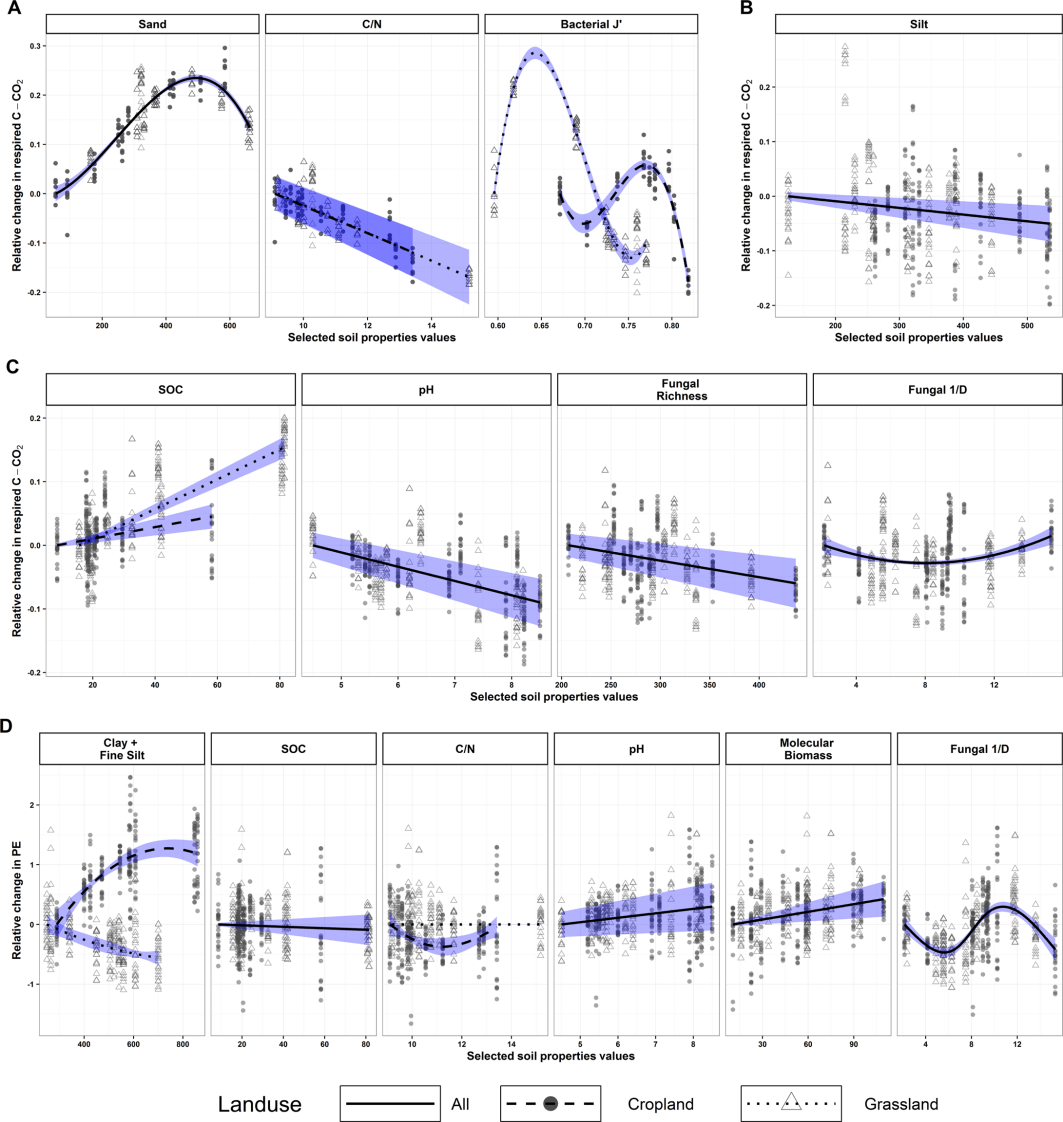
Selected soil properties and associated estimated relationships for each mineralisation parameter. (A) Control soil mineralisation (R_s,control_), (B) residue mineralisation (R_r_), (C) amended soil mineralisation (R_s,amended_) and (D) priming effect (PE). The x-axis represents the selected soil property values, and the y-axis (dimensionless) represents the relative change in the mineralisation parameter when selected soil property values vary. Units of soil properties are [g.kg^-1^ soil] for texture variables and soil organic carbon (SOC) content, [μg DNA.g^-1^ soil] for molecular biomass, [number of operational taxonomic units (OTUs)] for fungal richness, [pH unit] for pH and dimensionless for C:N ratio, bacterial evenness (J’) and fungal inverse Simpson (1/D). To facilitate reading, the relative change in the mineralisation parameter equals zero when the selected soil properties are at their minima. Solid black lines represent estimated relationships between the mineralisation parameter and the selected soil properties, regardless of land use (no interaction). Dashed and dotted black lines represent estimated relationships for cropland and grassland, respectively. Blue areas indicate the 95% confidence interval of the estimated relationships. Dots (cropland) and triangles (grassland) represent partial residuals, i.e. residuals of the models when all selected soil properties are accounted for except for the one considered.

All selected soil properties were significant in the models, except SOC content in PE models (P = 0.45) (Table 1). Among selected abiotic soil properties, texture covariates were selected for three models, but with a high relative importance only for R_s,control_ (8%), which was positively related to sand content (Fig 4). SOC content in interaction with land-use history was highly positively related to R_s,amended_ (13%), while the C:N ratio in interaction with land-use history was negatively related to R_s,control_ variability (7%). Soil pH was selected in models for R_s,amended_ and PE, explaining 12% and 13%, respectively; however, it was negatively related to R_s,amended_ and positively related to PE.

Among selected biological soil properties, molecular microbial biomass was influential only for PE, with a positive relationship and a low relative importance (3%). None of the diversity indexes were selected for R_r_. In control incubation, a polynomial relationship with bacterial J' in interaction with land-use history explained 44% of R_s,control_ variability. The relative importance of microbial diversity indexes in other models was lower: fungal 1/D, associated with a polynomial function, explained 9% of PE variability and less than 1% of that in R_s,amended_. Fungal richness was negatively related to R_s,control_ and explained 4% of its variability.

## Discussion

### Quality of the selected models

Except for bacterial 1/D, bacterial J’ and silt content, there was as much variability in soil property values and mineralisation response variables within each set of soils (cropland and grassland) as between the two sets, indicating that differences in the relationships built by the GAM approach for cropland and grassland were due more to differences in processes driving C dynamics between the land-uses than to differences in soil properties. However, the low power of the ANOVA–due to a small dataset (n = 10 for each set)–decreased its ability to detect significant differences. Grasslands tended to have higher and more variable SOC contents than croplands [39] and higher values of bacterial diversity indexes (H', J', 1/D). In terms of functioning, PE tended to be slightly higher in croplands, which agrees with a recent study showing an increase in PE intensity with an increase in land-use intensity [9].

Much of the variability was explained by the selected soil properties, as demonstrated by the percentage of deviance explained by each model. Thus, the soil properties contained enough information to explain SOC mineralisation. However, predictive quality depended more on the model. Not surprisingly, predictive quality was linked to model complexity, as assessed by its degrees of freedom (Table 1): the simpler the model, the higher was its predictive quality. The model for PE was the most complex but had the lowest quality, suggesting that processes involved in PE are also complex or that more relevant covariates, such as mineral nutrient availability [40,41], were not included.

### Relevance of microbial diversity indexes in explaining SOM dynamics

Microbial diversity indexes were selected for three activities describing SOM dynamics, demonstrating their ability to explain variability in SOM dynamics. The absence of microbial diversity indexes in the residue mineralisation model did not agree with results of a previous study [9], which observed a large importance of fungal richness using the same substrate. Indeed, on complex substrates such as wheat, the influence of microbial diversity may increase [42]. However, in our study, fungal communities may have been sensitive to the drying and long storage of soils [43]. Also, Tardy et al. [9] described mineralisation as the area under the curve of CO_2_ emissions, which captured mineralisation dynamics throughout the incubation period. As time is no longer needed in this model of mineralisation, other explanatory covariates can be detected more easily. The time variable was able to explain nearly all the variability in the residue mineralisation rate. In other words, variability in the residue mineralisation rate at each sample time was relatively small (coefficient of variation = 4–14%), likely due to using only one type of residue, and residue quality has been shown as an important driver of residue mineralisation [44].

Control soil mineralisation was explained better by bacterial diversity, while amended soil mineralisation and PE were explained better by fungal diversity. This difference can be explained by the observed activation of the fungal community in amended soil, leading to a PE [41,45] through successional changes in microbial community structure, with a gradual increase in the fungal:bacterial biomass ratio [46]. However, while the relative importance of microbial diversity was high in the control soil (44%), it tended to be lower in amended soil (4%) and for the PE (9%). Thus, while microbial diversity indexes can explain basal respiration, they are less able to explain respiration occurring from soil amended with an organic substrate or the PE, suggesting that both activities may depend more on microbial composition than on taxonomic richness and evenness [9,41,47]. When diversity indexes are replaced by abundances of bacterial and fungal phyla, and the same selection procedure is applied, model quality (according to Bayesian Information Criterion (BIC)) increases for the amended soil mineralisation and PE models but decreases for the other two (S3 Table). In the PE model, 24% of the variability was explained by phyla abundances, compared to only 9% when microbial diversity indexes are used. This highlights that processes related to PE may be driven more by microbial composition than diversity indexes.

For control soil mineralisation, bacterial evenness was the microbial diversity index selected. As stated by Hooper et al. [10], a relationship between microbial diversity and soil processes can be explained by i) an increased likelihood that key species will be present when richness increases or ii) by an increase in positive interactions (complementarity or facilitation) when diversity increases that increases rates of ecosystem processes. While indexes that include richness (richness, H’ and 1/D) can encompass both hypotheses, evenness considers only the relative abundance of each microbial group, regardless of the number of groups, and can capture only the second hypothesis. This suggests that greater equity in the abundance of microbial groups would increase these positive interactions. However, since all bacterial diversity indexes are strongly correlated with each other (S1 Fig), it is difficult to ensure that one index selected would have the capacity to explain variability in C dynamics better than the others.

Overall, we showed that microbial diversity indexes can be relevant covariates to include in SOM dynamics models, but that distinctions exist between bacteria and fungi and between different C-substrate pools. Systematically using C-labelled residues and distinguishing between bacterial and fungal diversity in future studies may represent a promising approach to (i) understand the complex relationship between microbial diversity and C mineralisation and (ii) define a suitable strategy for integrating microbial-diversity parameters in SOM modelling. Most current models include at least two compartments to model C dynamics of residues and SOM differently, which improves the prediction of overall C dynamics [4]. Due to differences in physiology, bacterial and fungal communities may influence C dynamics differently, whether individually [16] or in interaction [17,48].

### Implications for integrating microbial diversity in SOM dynamics models

Perveen et al. [14] developed a promising SOM dynamics model that represents two functional groups of microorganisms to account for interactions between residue and soil mineralisation (i.e. the PE). One major limitation of this kind of model is the difficulty in identifying groups given the current level of knowledge about the influence of microbial composition on soil processes. Hence, this model remains theoretical until knowledge improves. Since microbial diversity indexes appear to explain soil decomposition/mineralisation well, but composition appears to explain PE better, i) in models integrating processes involved in the PE, microbial composition likely better represents the influence of microbial communities, while ii) in models focusing only on decomposition/mineralisation processes, microbial diversity indexes may represent well the influence of microbial diversity on C dynamics.

Observing relationships between microbial diversity indexes can help to understand the influence of microbial diversity on C dynamics. Interestingly, the relationship between bacterial diversity and control soil mineralisation seemed to depend on land-use history. These relationships for both cropland and grassland were polynomial curves that overlapped little. Grassland soils seemed to be associated with high mineralisation rates and low bacterial evenness, while the opposite was observed for cropland soils. Consequently, land-use history and bacterial evenness appear to have confounding effects. This highlights one limitation of the method: when there is a strong relationship between covariates, it is difficult to know which one influences the other. In this case, it is more likely that land-use management has changed soil properties, such as bacterial diversity or SOC content, with consequences on C dynamics. Thus, we assume that a negative relationship exists between bacterial diversity and basal soil mineralisation. This is consistent with the negative relationship between fungal richness and amended-soil mineralisation. Such negative relationships were unexpected, as previous studies demonstrated positive relationships [6,7,49]. Nevertheless, the negative relationship does not necessarily contradict these studies, which focused on total flux. Respiration can decrease due to a decrease in the decomposition rate of the substrate or an increase in the substrate-assimilation yield of microorganisms. Hypotheses exist in the literature about the influence of microbial diversity on C assimilation yield [18]. C assimilation yield represents how microorganisms control the fate of soil C, i.e. the C is either used for bacterial growth and production of microbial products, such as exoenzymes or polysaccharides, or it is mineralised [50]. As patterns of allocation and production vary among microorganisms, the positive relationship between C-assimilation yield and microbial diversity may be due to the presence of species that efficiently assimilate the C to produce microbial products in species-rich communities. Beyond the individual level, this relationship may also result from an increase in positive interactions between microorganisms that leads to higher C assimilation yield overall: niche partitioning [10], complementarity between enzyme producers and “cheaters” i.e. microorganisms that benefit from decomposition products [51], leading to syntrophic relationships [52]. Predictions of SOM dynamics models have been highly sensitive to the C assimilation yield parameter [18]. The hypothesis of increased C assimilation yield with increased microbial diversity is of great interest, as it may help to increase model accuracy by calculating assimilation as a function of soil microbial diversity. Moreover, better knowledge of this relationship could help to understand soil’s response to climate warming, as it depends on C assimilation yield [53].

### Strengths and limitations of the method

As we raised hypotheses from relationships generated by the method, studying relationships between well-known abiotic soil properties and C dynamics was an appropriate way to investigate the method’s ability to build consistent relationships. First, the properties selected were consistent with those the literature. Overall, SOC explained 13% of C mineralisation in amended soil. It is well established that the amount of SOC drives the amount of soil C which is mineralised [43,54]. SOC content positively affected mineralisation, and the difference between this relationship in croplands and grasslands likely resulted from the difference in the dispersion of SOC content. In addition to the quantity of C substrates, the quality of SOM, estimated by the C:N ratio, appeared as an important factor controlling control soil C mineralisation [43]. The negative relationship may be related to stoichiometric needs of microorganisms for nutrients and limits to nutrient availability. Selection of soil pH for amended soil mineralisation and PE confirmed its importance for predicting C mineralisation. Soil pH is a well-known factor influencing C dynamics through its impact on the biomass [55], composition [56,57], structure and activity [58,59] of soil microbial communities. In other respects, the influence of pH on N dynamics [60,61] and other soil chemical properties is also well established [55,59,62]. Overall, these results demonstrate that the method enabled selecting relationships between mineralisation and certain soil properties already known to influence SOM dynamics. This is a good indicator that the method is able to select drivers of mineralisation and build consistent relationships.

We were unable to interpret some irregular relationships, however, such as the opposite relationships between texture and PE in croplands and grasslands. This difference is likely due to overfitting of data, to which GAM is sensitive, rather than to true biophysical relationships. In a dataset in which little is controlled, confounding among covariates is likely. For example, in the control-soil mineralisation model, one could argue that confounding occurs with SOC content because it is negatively correlated with bacterial evenness (S1 Fig). Forcing the model to include SOC content produced a model similar to the one for amended-soil mineralisation, but with much lower quality (BIC = -78 instead of -235, results not shown). Following these observations and comments by Burnham and Anderson [63], there are three major limitations of the approach used:

1. Results could be in a local minimum: the selection procedure would take too much time to test all possible models and cannot prevent models from falling into local minima. In practice, the model obtained cannot be improved but is not the best of all possible models.
2. There is no such thing as a "best model": the selection procedure improves models by optimising a criterion (in this study, the MSEP) at each step. In practice, it is likely that several models have similar criterion values and that the model selected is not significantly better than others. Consequently, models with similar quality may exist.
3. As stated, since relationships were constructed statistically, biophysical interpretations of them may not exist.

Consequently, despite the strengths of the method, the relationships it identified should be considered with caution. Future studies should confirm or disconfirm these relationships and raised hypotheses should be tested in more experimental studies.

## Conclusion

By using an innovative statistical data-mining approach on a dataset combining a broad sample of studied soils with high variability in soil properties and an incubation experiment monitoring C dynamics according to C source, we aimed to i) investigate whether microbial diversity indexes could help explain variability in C dynamics, ii) construct quantitative relationships between these indexes and variables describing C dynamics and iii) assess implications of these relationships for integrating microbial diversity in SOM dynamics models.

Despite some limitations of the approach, by monitoring SOM and residue mineralisation independently, we demonstrated that microbial diversity indexes could constitute good covariates to integrate in SOM dynamics models, depending on the C source and the processes considered. Specifically, microbial diversity indexes can help explain soil decomposition/mineralisation, while the PE seemed to be associated much more with microbial composition. Thus, we suggest two complementary approaches for future research. First, continue ongoing investigation of relationships between microbial composition, functional traits and soil C dynamics. More detailed knowledge should help to better represent microbial diversity and its role in mechanistic SOM dynamics models, especially those including the PE. Second, we argue that including microbial diversity indexes in mechanistic models could be as pertinent as including microbial composition. Quantitative relationships provided by our approach can help discover ways to do the latter. We suggest further investigation of the relationship between microbial diversity indexes and C-assimilation yield. One possibility is that the C-assimilation yield parameter could be modulated as a function of microbial diversity indexes in models.

## Supporting Information

S1 Fig Correlation matrix between soil properties. Upper part of the matrix: Pearson correlation coefficients. Size of figures is proportional to the absolute value of the coefficient. Lower part of the matrix: scatter plots between soil properties. Solid red lines represent smoothed estimates of the relationship between soil properties.
(TIFF)

S1 Table Information about soil sample locations. AMT, Average Monthly Temperature; AMP, Average Monthly Precipitation; AMETP, Average Monthly Evapotranspiration. ^a^ Coordinates follow the Lambert-93 projection.
(XLSX)

S2 Table Minimal dataset of response variables and covariates used in the study. AMT, Average Monthly Temperature; AMP, Average Monthly Precipitation; AMETP, Average Monthly Evapotranspiration; SOC, Soil Organic Carbon; C/N, soil Carbon/Nitrogen ratio; H', Shannon index; J', Evenness; 1/D, inverse Simpson index; R_s_, Respiration rate during control (R_s,control_) and amended (R_s,amended_) soils mineralisation; R_r_, Respiration rate during residue mineralisation. Coordinates (longitude and latitude) follow the Lambert-93 projection.
(XLSX)

S3 Table Comparison of quality between microbial diversity index-based models and phyla-based models. nVar, Total number of selected covariates; df, degrees of freedom; BIC, Bayesian Information Criterion; nDiv, Number of selected microbial diversity covariates (indexes or phyla abundance); %Div, Total percentage of variance explained by all selected microbial diversity covariates. ^a^ Models selected with microbial diversity indexes as potential microbial diversity covariates. ^b^ Models selected with phyla abundance as potential microbial diversity covariates. ^c^ Differences between BICs of phyla abundance-based models and microbial diversity index-based models. A positive value means a model based on phyla was better, while a negative value means a model based on diversity indexes was better.
(XLSX)
