# Supplementary figures and images for: Microbial Diversity Indexes Can Explain Soil Carbon Dynamics as a Function of Carbon Source
Source: PLoS One. 2016 Aug 23;11(8):e0161251. doi: 10.1371/journal.pone.0161251 (PMC4995005; doi:10.1371/journal.pone.0161251)

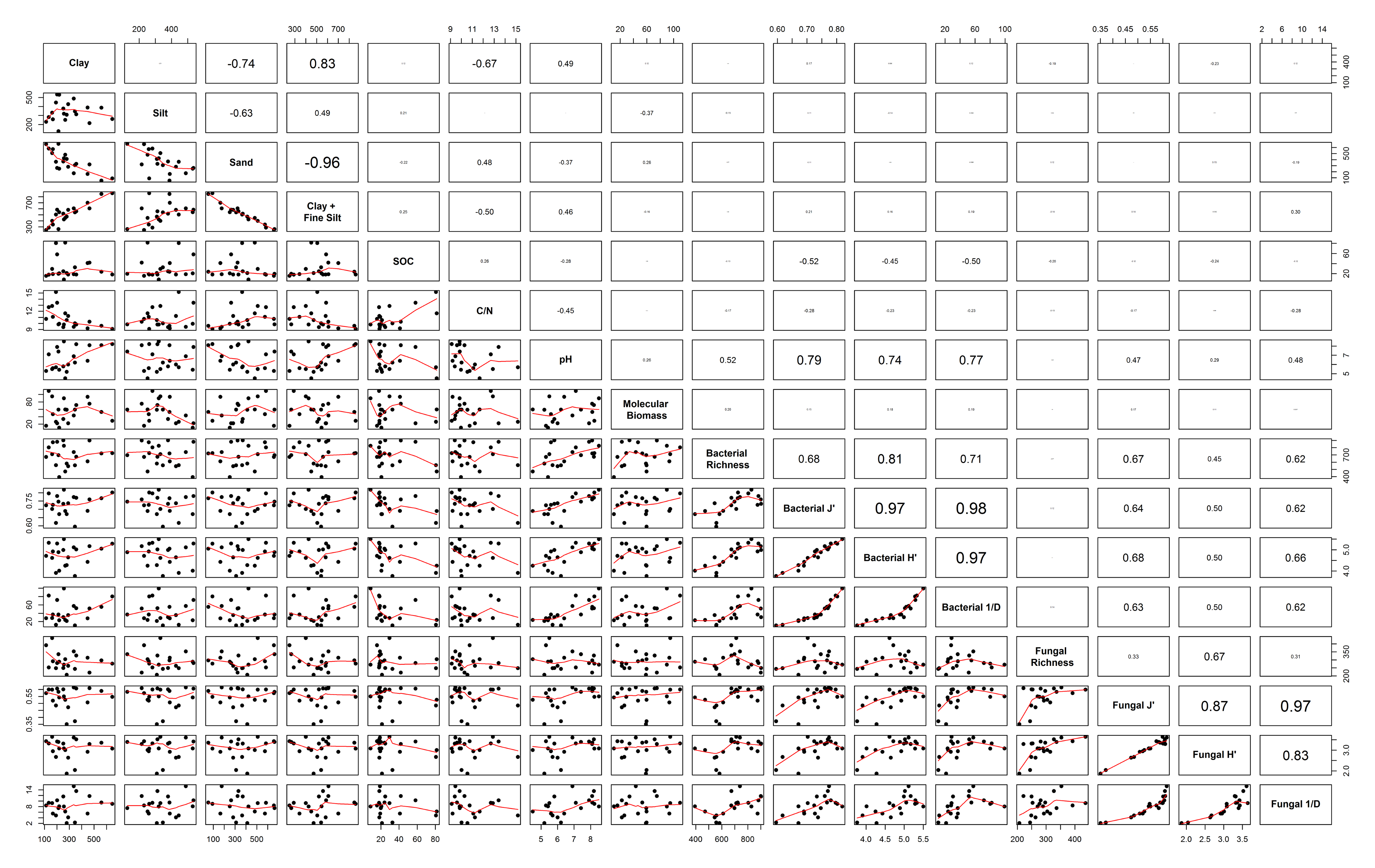

Supplement: S1 Fig — Upper part of the matrix: Pearson correlation coefficients. Size of figures is proportional to the absolute value of the coefficient. Lower part of the matrix: scatter plots between soil properties. Solid red lines represent smoothed estimates of the relationship between soil properties. (TIFF) [file pone.0161251.s001.tiff]
